# Isothermal Crystallization Kinetics and Their Effect on the Molding Process and Mechanical Properties of PAEK and PEEK

**DOI:** 10.3390/polym17192713

**Published:** 2025-10-09

**Authors:** Jindong Zhang, Kun Yu, Yunfeng Luo, Weidong Li, Xiangyu Zhong, Gang Liu, Jianwen Bao, Chunhai Chen

**Affiliations:** 1 National Key Laboratory of Advanced Composites, AVIC Composite Technology Center, AVIC Composite Corporation Ltd., Beijing 101300, China; zhangjindong@buaa.edu.cn (J.Z.); luoyf@avic.com (Y.L.); liwdhappy@163.com (W.L.); xyzhong2003@sohu.com (X.Z.); 2 State Key Laboratory of Advanced Fiber Materials, Center for Advanced Low-Dimension Materials, College of Materials Science and Engineering, Donghua University, Shanghai 201620, China; 1222717@mail.dhu.edu.cn (K.Y.); cch@dhu.edu.cn (C.C.); 3 Advanced Technology and Equipment Research Institute, Beijing University of Chemical Technology, Beijing 100029, China

**Keywords:** poly(aryletherketone) (PAEK), poly(etheretherketone) (PEEK), isothermal crystallization kinetics, molding process, tensile properties

## Abstract

The crystallization behavior of poly(aryletherketone) (PAEK) determines its applicable molding process and profoundly affects its mechanical properties. However, research on the crystallization behavior of new PAEKs and their impact on performance is still insufficient. In this work, the isothermal crystallization behavior of a novel PAEK was studied and compared with that of standard poly(etheretherketone) (PEEK). The influence of molding temperatures on the mechanical properties of thermoplastics was revealed by controlling the crystallization temperatures and analyzing the crystallization behavior. The results indicate that due to the disruption of the molecular structure regularity of PAEK, its melting temperature for primary crystallization is generally about 30 °C lower than that of PEEK, which is beneficial for its molding at lower temperatures. At the same undercooling level, the crystallization rate of PAEK is lower than that of PEEK, making it easier to control the crystallinity of PAEK through process parameters. The crystallinity of the thermoplastics increases with the increase in soaking time, thereby improving their tensile strength and modulus. The maximum crystallinity of PAEK is approximately 20.5%, which is lower than PEEK’s value of 31.8%. Therefore, under the same undercooling condition, the tensile strength and modulus of PEEK increase by up to 29.5% and 17.1%, respectively, compared to PAEK. Therefore, by precisely controlling the molding process parameters of PAEK, their crystallization behavior can be managed, enabling the achievement of various properties as needed.

## 1. Introduction

High-performance thermoplastic composites have become ideal materials in cutting-edge fields such as aerospace, nuclear energy, and new energy vehicles [1,2,3] due to their excellent mechanical properties, heat resistance, radiation resistance, and recyclability [4,5,6]. Among them, poly(aryletherketone) PEEK is the most widely used matrix in the poly(etheretherketone) PAEK family. Its carbon fiber-reinforced composites have a higher specific strength than aviation aluminum alloy [7]. Moreover, their outstanding toughness and extremely molding efficiency are unmatched by traditional thermosetting composites [8,9,10].

Although PEEK offers excellent comprehensive performance, its high melting temperature and viscosity result in processing temperatures generally above 380 °C, which significantly increases energy consumption and process complexity [11]. To address this issue, poly(etherketoneketone) (PEKK) or low-melting-temperature PAEK (LM-PAEK) is increasingly used as an alternative. The new types of PAEKs can enhance processing efficiency while maintaining heat resistance through molecular structure design. PEKK introduces asymmetric ketone groups into its molecular chain, lowering its melting temperature by 10–30 °C compared to PEEK and enhancing the processability [12]. LM-PAEK adjusts the ether ketone ratio in the molecular chain and the substitution point of the benzene ring to further lower the melting temperature, making it suitable for efficient molding technologies such as automatic fiber placement (AFP) and 3D printing [13,14]. Wang et al. [15] utilized the thermo-stamping process to fabricate L-shaped corners from LM-PAEK thermoplastic composites, investigating the effects of processing parameters on the quality of the components. The results show that the corners can be fabricated at 360 °C, and the processing time takes only 90 s to achieve high molding quality and curved beam strength. Consequently, PAEK has demonstrated significant advantages in enhancing the processing efficiency and mechanical properties of thermoplastic composites.

The mechanical properties of thermoplastics heavily rely on their crystallization kinetics, which are influenced by the molecular chain structure and external factors, especially temperature. Pérez-Martín et al. [16] studied the influence of non-isothermal cooling cycles on the extent of crystallization. The Velisaris–Seferis model was implemented, and a correlation between the isothermal holding temperature and the crystallization rate constants was established. According to the results, optimum isothermal hold temperatures during cooling for fabricating CF/PEKK composites are determined. Choupin et al. [17] studied the crystallization kinetics of PEKK with different terephthalic/isophthalic (T/I) ratios. The results show that PEKK with a higher T/I ratio has faster crystallization kinetics than that with a lower T/I ratio. However, faster crystallization kinetics enhance the melting temperature and viscosity of the PEKK, which requires higher processing temperatures. Gao et al. [18] studied the variation in tensile properties of PEKK and PEEK with crystallinity by controlling the cooling rate. The results indicate that higher crystallinity increases the stiffness of the thermoplastics. However, most research studying the impact of crystallization on performance focus primarily on PEEK and PEKK, with limited research on LM-PAEK, despite its great potential for application. The ordered arrangement and thicker layers of crystals can hinder the slip between them. Due to the significant differences in the molecular structure of PAEKs, their crystallization characteristics, such as nucleation rate, crystallinity, and spherulite morphology, have a more complicated effect on their mechanical properties. In addition, current research on crystallization kinetics primarily focuses on non-isothermal crystallization [19,20], as traditional thermoplastic composite molding processes, such as compression molding, occur during a cooling process. However, in high-efficiency molding methods such as injection molding of thermoplastics or stamping of thermoplastic composites, the melt often rapidly contacts a low-temperature mold, causing the temperature quickly decreases to the mold temperature [15,21]. As a result, crystallization during the cooling process accounts for only a small portion, and the role of isothermal crystallization cannot be ignored. However, for PAEK, which exhibits complex secondary crystallization behavior, the classical Avrami equation cannot accurately describe the entire crystallization process. Hence, the Velisaris–Seferis equation is widely used in the literature to address this challenge [16,17,22,23].

In summary, further research is necessary to explore the isothermal crystallization behavior of emerging LM-PAEKs and the correlation with their macroscopic properties. Therefore, in this work, the isothermal crystallization behavior of PAEK and PEEK was studied using DSC testing based on the Velisaris–Seferis model. The differences in crystal morphology between them were observed using a polarizing microscope at various soaking times under the same undercooling conditions. Finally, the tensile properties of the thermoplastics, injection-molded with different soaking times under the same undercooling conditions, were tested. The results are crucial for understanding the relationship between polymer structure and properties, as well as guiding the molding process of high-performance thermoplastics and their composites.

## 2. Materials and Methods

### 2.1. Raw Materials

**Figure 1:**
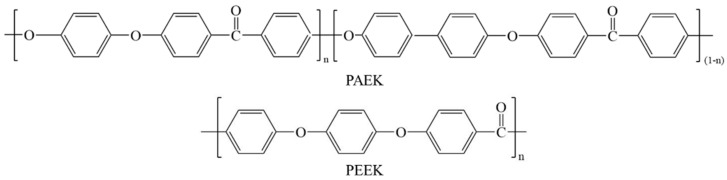
The molecular structure of PAEK and PEEK.

PAEK (brand PAEK-L) and PEEK (brand PEEK-90) were provided by Hairuite Engineering Plastics Co., Ltd., Jiamusi, China. in the form of fine powder and grain. Their molecular structure is shown in Figure 1. The glass transition temperatures of PAEK-L and PEEK-90 are both about 146 °C. The melt flow rate (MFR) of these materials is about 90 g/10 min (at 380 °C with 5 kg pressure).

### 2.2. Differential Scanning Calorimetry (DSC) Testing

**Figure 2:**
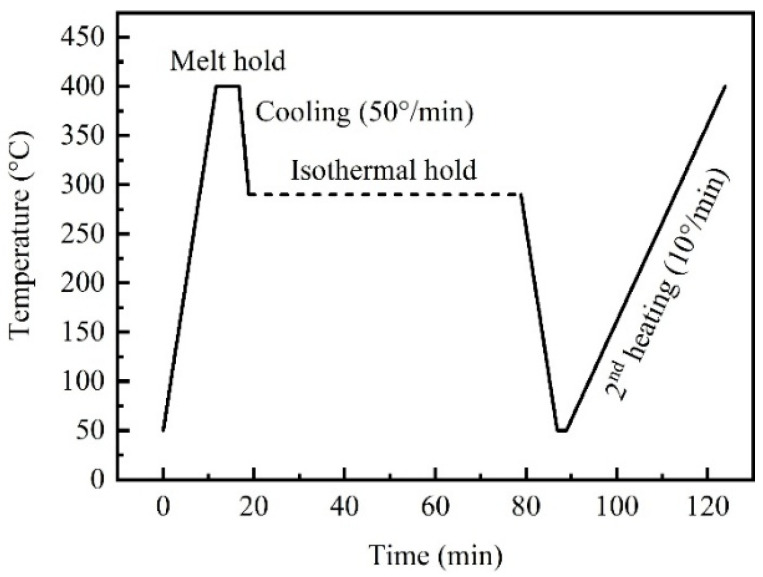
Testing conditions of DSC.

Isothermal crystallization behavior tests were conducted by DSC (DSC250, TA, Newcastle, DE, USA). The PEEK and PAEK samples are in the form of fine powder, with a weight of 5–7 mg. The testing used a sealed aluminum crucible under a nitrogen atmosphere at a flow rate of 50 mL/min. The thermoplastic samples were heated to 400 °C at a rate of 20 °C/min and held for 10 min to erase thermal history. The isothermal test was conducted from the melting temperature to five different isothermal temperatures at a cooling rate of 50 °C/min. Among them, the isothermal temperatures of PAEK samples were 260 °C, 270 °C, 275 °C, 280 °C and 285 °C, while those of PEEK samples were 290 °C, 300 °C, 305 °C, 310 °C and 315 °C. The samples were held at isothermal temperatures for 60 min before being cooled to 50 °C. After crystallization, the samples were heated again to 400 °C at a rate of 10 °C/min to analyze the effects of different crystallization temperatures on the glass transition temperature, melting temperature, and crystallinity of the samples. The testing conditions are shown in Figure 2.

### 2.3. Isothermal Crystallization Kinetics Model

The Avrami equation is a classic universal equation for studying the crystallization kinetics of polymers [24,25]:

(1) $$\alpha (t)=1-\exp(-kt^{n})$$

where k is the crystallization rate constant, n is the Avrami exponent, α(t) is the relative crystallinity, which is defined as the change in enthalpy of crystallization over time at a specific temperature. It can be expressed as follows:

(2) $$\alpha \left(t\right)\;=\;\int_{0}^{t}\mathrm{dQ}(t)dt/\int_{0}^{\infty }\mathrm{dQ}(t)dt$$

where dQ(t) is the heat flux, $\int_{0}^{t}\mathrm{dQ}(t)dt$ is the crystallinity at time t, $\int_{0}^{\infty }\mathrm{dQ}(t)dt$ is the maximum crystallinity at a specific temperature.

Taking the logarithm on both sides of the Avrami equation:

(3) $$\ln\left[-\left(1-\ln\left(1-\alpha (t)\right)\right)\right]\;=\;\ln k\;+\;n\ln t$$

Therefore, based on the heat flux results of isothermal DSC tests, curves of $\ln\left[-\left(1\;-\ln\left(1-\alpha (t)\right)\right)\right]$ versus lnt can be ploted and linear fitted. The slope and intercept of the fitting equation are n and lnk, respectively.

However, extensive research has found that the Avrami equation cannot be fully applicable to the entire isothermal crystallization process involving secondary crystallization. Due to the different mechanisms and crystal morphologies of primary and secondary crystallization, the n value of secondary crystallization is often lower. Thus, the functional relationship between $\ln\left[-\left(1\;-\ln\left(1-\alpha (t)\right)\right)\right]$ and lnt throughout the crystallization process is approximately bilinear, which cannot be described by a single Avrami equation.

A solution to this problem is the Velisaris–Seferis model, which superimposes two independent Avrami equations corresponding to the primary and secondary crystallization processes, respectively [16,17]:

(4) $$\alpha \left(t\right)\;=\;w_{1}\left(1\;-\;\exp\left(-k_{1}t^{n_{1}}\right)\right)\;+\;w_{2}\left(1\;-\;\exp\left(-k_{2}t^{n_{2}}\right)\right)$$

where $w_{1}$ and $w_{2}$ are the weight factors for primary and secondary crystallization, respectively, satisfying a fixed functional relationship of $w_{1}\;+\;w_{2}\;=\;100\%$. $k_{1}$ and $k_{2}$ are the rate constants for primary and secondary crystallization, respectively.

The total crystallinity (χ) of the thermoplastic is calculated according to the following formula:

(5) $$\chi \;={\;\Delta H}_{m}/{\Delta H}_{t}$$

where ${\Delta H}_{m}$ is the melting enthalpy, ${\Delta H}_{t}$ is the theoretical melting enthalpy of complete crystallization of PAEK, taken as 130 J/g [26]. It should be noted that due to the different structures of PAEKs, there will inevitably be some differences in their theoretical melting enthalpy of complete crystallization. However, considering the difficulty of measuring theoretical melting enthalpy and the convenience of visually comparing the crystallinity of different structures of PAEKs, 130 J/g is generally adopted for PEEK, PEKK, and other structures of PAEKs in the literature [27,28].

### 2.4. Microscope AnalysisTensile Testing

The isothermal crystallization behavior of the thermoplastics was observed by a polarizing microscope (DM4 P, Leica, Wetzlar, Germany). Around 1 mg of thermoplastic powder was evenly scattered between two microscope slides. Then, the slides were placed on a hot stage (THMS600, Linkam, Salfords, UK). The hot stage was heated to 400 °C for 10 min to form a thin thermoplastic film. The PAEK and PEEK film was cooled down to 285 °C and 315 °C, respectively, holding for 60 min for observation.

### 2.5. Specimens Preparation

**Figure 3:**
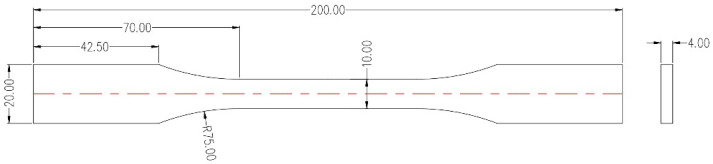
Specific dimensions of the tensile testing specimen.

PAEK and PEEK tensile specimens were prepared by an injection machine (TY-600, TAYU, Dongguan, China). The melting temperatures of PAEK and PEEK were set to 360 °C and 380 °C, respectively, and the mold temperatures were set to 285 °C and 315 °C. After the thermoplastic was injected into the mold, the temperature was held for 0 min, 3 min, 7 min, and 15 min. Then, the specimens were quickly demolded and cooled to room temperature. The sample is dumbbell-shaped, with specific dimensions as shown in Figure 3.

### 2.6. Tensile Testing

Tensile properties of the thermoplastic castings were tested by a universal mechanical testing machine (5982-100 kN, Instron, Boston, MA, USA) with standard GB/T 2567-2021 [29] as a reference. The loading rate was 2 mm/min, and the strain was recorded by a sight extensometer to calculate the modulus. At least five specimens were tested for each test, and the average value and the coefficient of variation (CV) were calculated.

## 3. Results and Discussion

### 3.1. Isothermal Crystallization Kinetics

**Figure 4:**
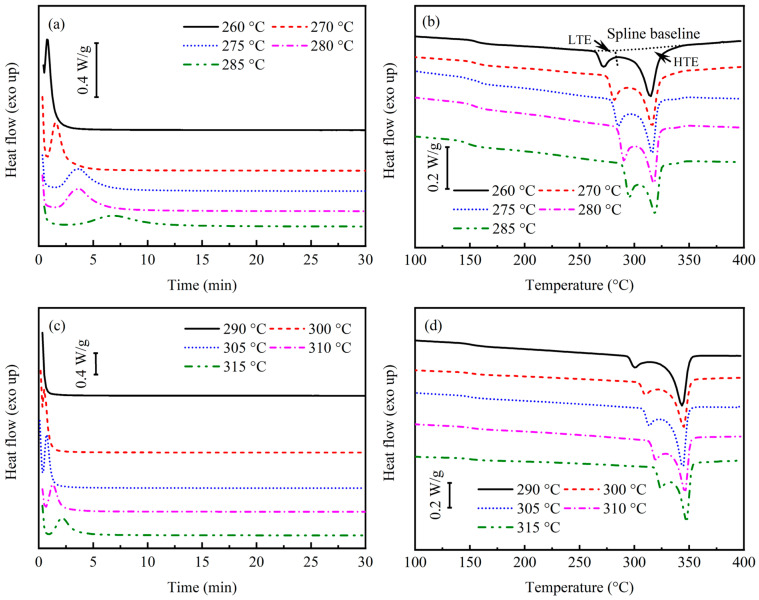
Heat flow curves and secondary heating curves of isothermal crystallization by DSC. (**a**) Isothermal heat flow curves of PAEK; (**b**) secondary heating curves of PAEK; (**c**) isothermal heat flow curves of PEEK; (**d**) secondary heating curves of PEEK.

The DSC heat flux curves and secondary heating curves for PAEK and PEEK crystallization at different isothermal temperatures are shown in Figure 4. The characteristic temperatures and melting enthalpy in the secondary heating curves are shown in Table 1. The ratio of low-temperature endotherm (LTE) to high-temperature endotherm (HTE) listed in the table was determined by fitting the baseline using the spline interpolation method, as shown in Figure 4b. Due to secondary crystallization, the secondary melting peaks of the thermoplastic after isothermal crystallization in Figure 4b,d are both bimodal. Among them, the LTE corresponds to the melting of secondary crystallization, while the HTE corresponds to the melting of primary crystallization. From Table 1, it can be observed that compared to PEEK, PAEK generally has a lower HTE peak temperature ($T_{H}$) of approximately 30 °C and lower crystallinity, which relates to differences in its molecular structure. Since PAEK molecules have less structural regularity than PEEK molecules, they hinder molecular movement and the orderly formation of crystal phases, which reduces crystallization potential and results in lower crystallinity and melting temperature [16,17,28].

For secondary crystallization, the LTE peak temperature ($T_{L}$) is generally about 10 °C higher than the isothermal crystallization temperature, indicating the mechanism of secondary crystallization. Secondary crystallization and primary crystallization are independent processes. Thus, secondary crystallization must rely on the formation of primary crystallization and grow in its gaps [16,30]. The LTE enthalpy (${\Delta H}_{L}$) to HTE enthalpy (${\Delta H}_{H}$) ratio (${\Delta H}_{L}:{\Delta H}_{H}$) in Table 1 gradually increases with the increase in isothermal crystallization temperature, indicating a corresponding increase in the proportion of secondary crystallization. The growth of spherulites is governed by two mechanisms: nucleation and growth. At lower temperatures, the nucleation rate surpasses the growth rate, resulting in more but smaller spherulites. At higher temperatures, the growth rate is influenced by the nucleation rate, leading to fewer but larger spherulites [17,30]. Consequently, at higher temperatures, the larger spherulites have more space, which benefits the growth of secondary crystals.

The difference in isothermal crystallization kinetics can be observed from the heat flux curves of Figure 4a,c at different isothermal temperatures. The main crystallization temperature range of PEEK is 290–315 °C. Additionally, only a partial crystallization peak appears at 290 °C, and the enthalpy of crystallization heat release cannot be calculated, indicating that most crystallization has already completed when the sample is cooled to the isothermal temperature. However, when the temperature exceeds 315 °C, the existence of crystal nuclei becomes unstable, and the heat flow curve cannot maintain equilibrium for a long time. For PAEK, the main crystallization temperature range is lowered to 260–285 °C. Meanwhile, PAEK demonstrates a slower crystallization rate than PEEK at the same undercooling. The reason remains related to changes in its molecular chain structure, which slows down the migration speed of some molecular chains arranged regularly.

**Figure 5:**
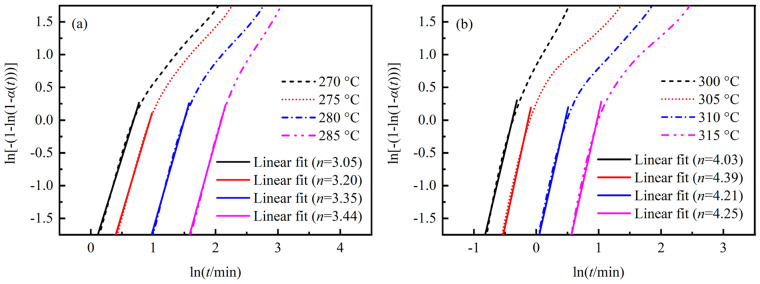
Plots of $\ln[-\left(1-\ln\left(1-\alpha (t)\right)\right)]$ versus lnt of isothermal crystallization. (**a**) PAEK; (**b**) PEEK.

According to the Avrami equation, linear fitting was performed on the primary crystallization process of PAEK and PEEK to determine the value of $n_{1}$, as shown in Figure 5. The $n_{1}$ values of PAEK are 3.05, 3.20, 3.35, and 3.44 at different isothermal temperatures, while those of PEEK are 4.03, 4.39, 4.21, and 4.25, respectively. According to the physical meaning of n, n = 3 indicates the three-dimensional growth of spherulites, and the result for PEEK being greater than 3 is currently considered to represent a more complex mode of spherulite growth [20]. It should be noted that it is generally believed that n is not affected by the crystallization temperature, so the numerical fluctuations here belong to experimental errors [16,31]. The $n_{1}$ mean ± standard deviation of PAEK is 3.26 ± 0.17, while that of PEEK is 4.22 ± 0.15. The coefficient of variation is as low as 5.2% and 3.6%, respectively. Thus, the fitting $n_{1}$ values of PAEK and PEEK are reliable, and the mean values can be applied for subsequent fitting. Moreover, it is believed that secondary crystallization is a two-dimensional layered structure between primary crystals. Therefore, $n_{2}$ is uniformly taken as 2 [16]. The known parameters were input into Equation (4) to determine the constant coefficients $k_{1}$ and $k_{2}$, and the residual sum of squares (RSS) was used to evaluate the fitting quality, as shown in Table 2. The RSS of each sample at different soaking temperatures is generally much less than 1, indicating a good fitting quality.

**Figure 6:**
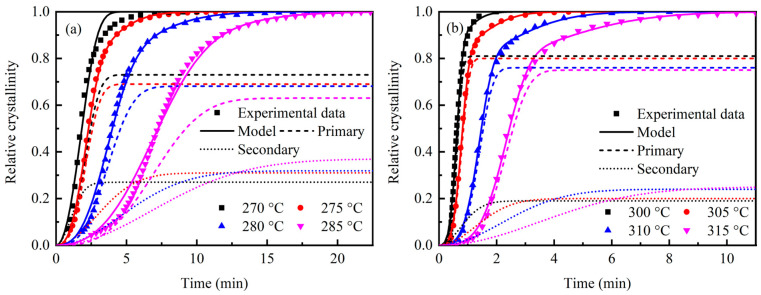
Fitting results of the isothermal crystallization kinetic model. (**a**) PAEK; (**b**) PEEK.

Based on the fitting parameters in Table 2, the model fitting curves for the relative crystallinity and time of PAEK and PEEK during isothermal crystallization at specific temperatures can be obtained. The comparison with experimental results is shown in Figure 6. The Velisaris–Seferis model effectively addresses the limitations of the Avrami equation in fitting the secondary crystallization process, and the fitting results closely match the experimental data.

### 3.2. Crystallization Behavior and Mechanical Properties

**Figure 7:**
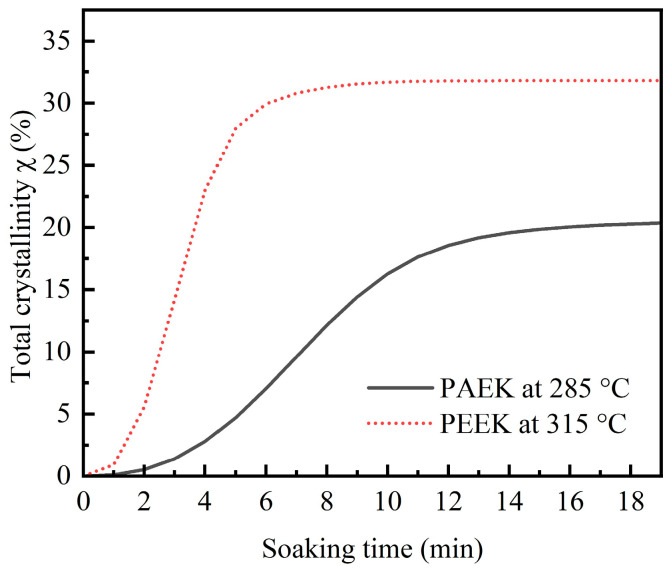
Total crystallinity versus soaking time of PAEK at 285 °C and PEEK at 315 °C.

Based on the calculation results of the Velisaris–Seferis model combined with the test results of total crystallinity, the variation in crystallinity over soaking time under the same undercooling conditions can be compared between PAEK and PEEK, as shown in Figure 7. According to the model, the total crystallinity of the thermoplastic also follows an exponential function over soaking time. At the same soaking time, the crystallinity of PEEK is always higher than that of PAEK. Additionally, under this undercooling condition, the maximum crystallinity of PEEK (~31.8%) is significantly higher than that of PAEK (~20.5%). This is also caused by the relatively regular molecular structure of PEEK, which enhances its crystallization rate and crystallinity.

**Figure 8:**
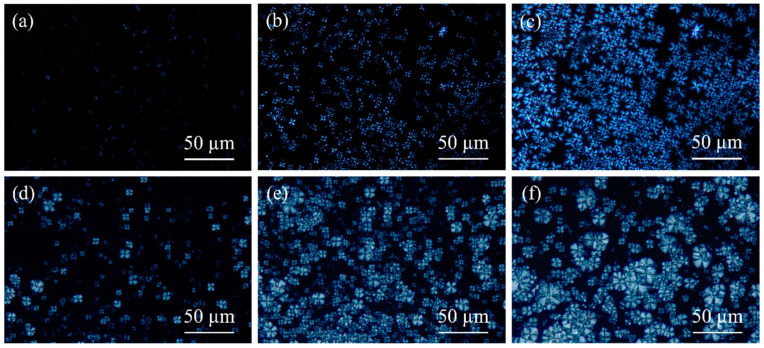
Polarized microscope images of isothermal crystallization of PAEK and PEEK at different soaking times. (**a**) PAEK at 285 °C for 3 min; (**b**) PAEK at 285 °C for 7 min; (**c**) PAEK at 285 °C for 15 min; (**d**) PEEK at 315 °C for 3 min; (**e**) PEEK at 315 °C for 7 min; (**f**) PEEK at 315 °C for 15 min.

The polarizing microscopy images of PAEK and PEEK isothermal crystallization are shown in Figure 8. The size and quantity of spherulites in PAEK (see Figure 8a–c) and PEEK (see Figure 8d–f) gradually increase with longer soaking time. This is because the crystals have a more time to grow, especially for the secondary crystallization. The secondary crystallization process occurs after the primary crystallization (as illustrated in Figure 6, the secondary crystallization curve lags behind the primary crystallization curve) and takes place in the intervals left by the primary crystallization. Consequently, a longer holding time results in larger crystal growth. At the same soaking time, the number and size of PEEK spherulites are always greater than those of PAEK. This further demonstrates that PEEK has a faster crystallization rate, enabling the formation of more crystal nuclei in the same amount of time and providing more time for crystal growth, which results in larger and more complete spherulites.

**Figure 9:**
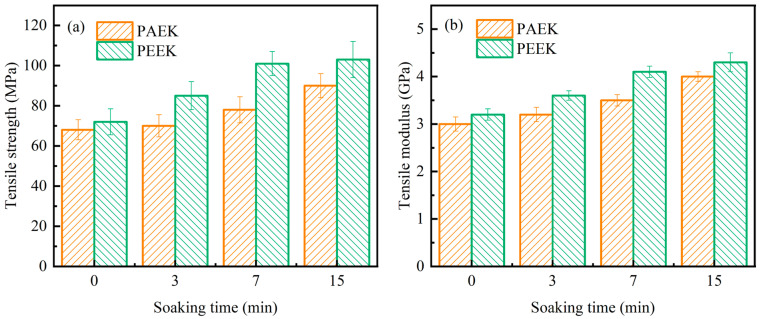
Tensile properties versus soaking time of PAEK at 285 °C and PEEK at 315 °C. (**a**) Tensile strength; (**b**) tensile modulus.

The tensile properties of PAEK and PEEK fabricated at different soaking times are shown in Figure 9. From Figure 9a, the tensile strength of PAEK fabricated at different soaking times is 68 ± 4.9 MPa, 70 ± 5.5 MPa, 78 ± 6.4 MPa and 90 ± 6.0 MPa, while that of PEEK is 72 ± 6.5 MPa, 85 ± 6.9 MPa, 101 ± 6.1 MPa and 103 ± 8.8 MPa. The tensile strength of the thermoplastics rises as the molding soaking time increases. This is caused by the rise in crystallinity, which can enhance the tensile strength of the thermoplastics [32]. Meanwhile, due to the higher crystallinity of PEEK compared to PAEK, the tensile strength of PEEK at each soaking time is greater by 5.9%, 21.4%, 29.5%, and 14.4%, respectively, than that of PAEK. From Figure 9b, the tensile modulus of PAEK fabricated at different soaking times is 3.0 ± 0.15 GPa, 3.2 ± 0.15 GPa, 3.5 ± 0.12 GPa and 4.0 ± 0.10 GPa, while that of PEEK is 3.2 ± 0.13 GPa, 3.6 ± 0.10 GPa, 4.1 ± 0.12 GPa and 4.3 ± 0.19 GPa. In addition, the tensile modulus of PEEK at each soaking time is greater by 6.7%, 12.5%, 17.1%, and 7.5%, respectively, than that of PAEK. The trend of change in the tensile modulus of the thermoplastics is similar to that of the tensile strength, indicating that the stiffness of the thermoplastic also increases with the increase in crystallinity. However, in our previous studies, we found that higher crystallinity and larger crystals negatively affect the ductility of PAEK [33]. Consequently, the impact of crystallinity on stiffness and toughness of PAEK is often in conflict. As the matrix of composites, increasing stiffness helps improve the in-plane mechanical properties (especially compression performance), but it reduces the matrix toughness, which harms the interlaminar fracture toughness and impact resistance of composites [33,34]. Therefore, when choosing the molding process parameters for thermoplastics and their composites, it is necessary to comprehensively consider their impact on the final mechanical properties of the products.s

## 4. Conclusions

To address the issue of high processing temperatures of PEEK, new structures of PAEKs continue to emerge. The performance of PAEK and its composites strongly depends on their bulk crystallization behavior. Therefore, research is urgently needed to investigate the impact of differences in structure on the crystallization behavior of thermoplastics and their mechanical properties. In this work, the isothermal crystallization behavior of a novel PAEK and its effect on the mechanical properties of the thermoplastic were studied and compared with the commonly used PEEK. The Velisaris–Seferis model was used to describe the isothermal crystallization behavior of the thermoplastics, and the results aligned closely with the DSC experimental results. The differences in crystal morphology of PAEK and PEEK were observed using a polarizing microscope at different soaking times under the same undercooling conditions. Finally, the tensile properties of the thermoplastics, injection-molded with different soaking times under the same undercooling, were tested. PAEK has a melting peak temperature that is approximately 30 °C lower than that of PEEK, making it advantageous to mold at lower processing temperatures. Due to the disruption of the structural regularity of PAEK, its crystallization kinetics are slower than those of PEEK. The maximum crystallinity of PAEK is approximately 20.5%, which is lower than PEEK’s value of 31.8%. Therefore, under the same undercooling condition, the tensile strength and modulus of PEEK increase by up to 29.5% and 17.1%, respectively, compared to PAEK. However, the increase in modulus negatively affects the ductility of the thermoplastic, leading to a decrease in the interlayer fracture toughness and impact resistance of its composites. These results contribute to understanding the relationship between the condensed structure and properties of thermoplastics. By precisely controlling the molding process parameters of PAEK and its composites, their crystallization behavior can be managed, enabling the achievement of various in-plane properties and interlaminar toughness as needed.

## Figures and Tables

**Table 1:** Characteristic temperature and enthalpy in isothermal crystallization secondary heating curves of PAEK and PEEK.

Isothermal Temperature (°C)	PAEK						
	$T_{g}$ (°C)	$T_{L}$ (°C)	${\Delta H}_{L}$ (J/g)	$T_{H}$ (°C)	${\Delta H}_{H}$ (J/g)	χ (%)	${\Delta H}_{L}:{\Delta H}_{H}$
260	155.3	271.5	6.0	314.9	24.7	23.6	19:81
270	154.6	281.8	8.9	316.4	23.8	25.2	27:73
275	154.3	285.7	8.3	316.5	18.8	20.8	31:69
280	153.8	290.8	10.3	318.0	22.1	24.9	32:68
285	146.5	295.5	9.9	319.4	16.7	20.5	37:63
	**PEEK**						
290	150.5	300.9	7.4	343.7	36.4	33.7	17:83
300	150.5	310.4	7.4	344.6	31.7	30.1	19:81
305	150.3	313.7	9.3	344.9	35.5	34.5	20:80
310	150.1	319.8	9.9	346.1	31.0	31.5	24:76
315	149.7	324.4	10.4	347.7	31.0	31.8	25:75

$T_{g}$: glass transition temperature. $T_{L}$: low-temperature endotherm peak temperature. ${\Delta H}_{L}$: low-temperature endotherm enthalpy. $T_{H}$: high-temperature endotherm peak temperature. ${\Delta H}_{H}$: high-temperature endotherm enthalpy. ${\Delta H}_{L}:{\Delta H}_{H}$: low-temperature endotherm to high-temperature endotherm ratio. χ: total crystallinity.

**Table 2:** Fitting parameters of isothermal crystallization kinetic model.

Isothermal Temperature (°C)	PAEK			
	$w_{1}$	$k_{1}$	$k_{2}$	RSS
270	0.73	0.07920	0.50754	0.06194
275	0.69	0.07471	0.06312	0.01008
280	0.68	0.01384	0.02374	0.02864
285	0.63	0.00201	0.01110	0.07235
	**PEEK**			
300	0.81	5.06146	0.92508	0.37935
305	0.80	1.92739	0.32118	0.00395
310	0.76	0.19232	0.10777	0.00404
315	0.75	0.02511	0.04199	0.00864

$w_{1}$: weight factor for primary crystallization. $k_{1}$: rate constant for primary crystallization. $k_{2}$: rate constant for secondary crystallization. RSS: residual sum of squares.

## Data Availability

Data is contained within the article.

## References

[1] Miranda R., Scalici T., Di Franco F., Santamaria M., Valenza A. (2024). Enhancing the PEEK composites-titanium interface performances through electrochemical treatment in fibre-metal laminates for aerospace applications. Int. J. Adhes. Adhes..

[2] Cao Y., Yang H., Wan K., Li D., He Q., Wu H. (2024). High-performance PEEK composite materials research on 3D printing for neutron and photon radiation shielding. Compos. Part A—Appl. S..

[3] Eko A.J., Epaarachchi J., Jewewantha J., Zeng X. (2025). A review of type IV composite overwrapped pressure vessels. Int. J. Hydrogen Energ..

[4] Zhang H., Zhang Z., Long Y., Ran X., Guo Y., Li Y. (2025). Enhanced mechanical properties of continuous carbon fiber-reinforced PEEK composites via process parameters optimization and assisted infrared irradiation heating. Compos. Commun..

[5] Nikonovich M., Ramalho A., Emami N. (2024). Effect of cryogenic aging and test-environment on the tribological and mechanical properties of PEEK composites. Tribol. Int..

[6] Dandy L.O., Oliveux G., Wood J., Jenkins M.J., Leeke G.A. (2015). Accelerated degradation of polyetheretherketone (PEEK) composite materials for recycling applications. Polym. Degrad. Stabil..

[7] Dai J.N., Kou S.Q., Yang H.Y., Xu Z.B., Shu S.L., Qiu F., Jiang Q.C., Zhang L.C. (2022). High-content continuous carbon fibers reinforced PEEK matrix composite with ultra-high mechanical and wear performance at elevated temperature. Compos. Struct..

[8] Quan D., Deegan B., Binsfeld L., Li X., Atkinson J., Ivanković A., Murphy N. (2020). Effect of interlaying UV-irradiated PEEK fibres on the mechanical, impact and fracture response of aerospace-grade carbon fibre/epoxy composites. Compos. Part B—Eng..

[9] Cao J., Jiang B., Li Z., Dang Z., Zhang C. (2024). Evaluating the loading rate dependency of mode I delamination for composite laminates at different temperatures. Compos. Sci. Technol..

[10] Chen H., Li S., Wang J., Ding A. (2021). A focused review on the thermo-stamping process and simulation progresses of continuous fibre reinforced thermoplastic composites. Compos. Part B—Eng..

[11] Driezen J., Herrmann A.S. (2023). Characterization and modelling of the crystallization behavior of PEEK for polymer processing techniques. Polymer..

[12] Arquier R., Iliopoulos I., Régnier G., Miquelard-Garnier G. (2022). Consolidation of continuous-carbon-fiber-reinforced PAEK composites: A review. Mater. Today Commun..

[13] Heathman N., Koirala P., Yap T., Emami A., Tehrani M. (2023). In situ consolidation of carbon fiber PAEK via laser-assisted automated fiber placement. Compos. Part B-Eng..

[14] Yi N., Davies R., Chaplin A., McCutchion P., Ghita O. (2021). Slow and fast crystallising poly aryl ether ketones (PAEKs) in 3D printing: Crystallisation kinetics, morphology, and mechanical properties. Addit. Manuf..

[15] Wang W., Yao J., Zhang J., Wang Q., Liu G., Wang M. (2025). High-performance CF/LM-PAEK L-shaped corners by the hot stamping technique with low stamping temperature. Polym. Compos..

[16] Pérez-Martín H., Mackenzie P., Baidak A., Brádaigh C.M.Ó., Ray D. (2022). Crystallisation behaviour and morphological studies of PEKK and carbon fibre/PEKK composites. Compos. Part A—Appl. S..

[17] Choupin T., Fayolle B., Régnier G., Paris C., Cinquin J., Brulé B. (2018). A more reliable DSC-based methodology to study crystallization kinetics: Application to poly(ether ketone ketone) (PEKK) copolymers. Polymer.

[18] Gao S.L., Kim J.K. (2000). Cooling rate influences in carbon fibre/PEEK composites. Part I. Crystallinity and interface adhesion. Compos. Part A—Appl. S..

[19] Li X., Zhao Y., Wang K. (2020). Interfacial crystallization behavior of poly(ether-etherketone) on polyimide-modified CCF300 carbon fibers. Polym. Compos..

[20] Yang X., Wu Y., Wei K., Fang W., Sun H. (2018). Non-isothermal crystallization kinetics of short glass fiber reinforced poly (ether ether ketone) composites. Materials.

[21] Wang W., Zhang J., Yao J., Liu G., Wang M. (2025). The effect of tool temperature and dwell time on the quality and CBS properties of L-shaped CF/LM-PAEK corners manufactured by the thermo-stamping process. J. Compos. Mater..

[22] Velisaris C.N., Seferis J.C. (1986). Crystallization kinetics of polyetheretherketone (peek) matrices. Polym. Eng. Sci..

[23] Hsiao B.S., Chang I.Y., Sauer B.B. (1991). Isothermal crystallization kinetics of poly(ether ketone ketone) and its carbon-fibre-reinforced composites. Polymer.

[24] Avrami M. (1939). Kinetics of phase change. I General theory. J. Chem. Phys..

[25] Avrami M. (1940). Kinetics of phase change. II Transformation-time relations for random distribution of nuclei. J. Chem. Phys..

[26] Panda J.N., Bijwe J., Pandey R.K. (2019). Optimization of the amount of short glass fibers for superior wear performance of PAEK composites. Compos. Part A—Appl. S..

[27] Audoit J., Rivière L., Dandurand J. (2019). Thermal, mechanical and dielectric behaviour of poly(aryl ether ketone) with low melting temperature. J. Therm Anal. Calorim..

[28] Cortés L.Q., Caussé N., Dantras E. (2016). Morphology and dynamical mechanical properties of poly ether ketone ketone (PEKK) with meta phenyl links. J. Appl. Polym. Sci..

[29] (2021). Test Method for Properties of Resin Casting Body.

[30] Pérez-Martín H., Mackenzie P., Baidak A., Brádaigh C.M.Ó., Ray D. (2021). Crystallinity studies of PEKK and carbon fibre/PEKK composites: A review. Compos. Part B—Eng..

[31] Dai G., Zhan L., Guan C., Huang M. (2021). The effect of cooling rate on crystallization behavior and tensile properties of CF/PEEK composites. J. Polym. Eng..

[32] Lee I.G., Kim D.H., Jung K.H., Kim H.J., Kim H.S. (2017). Effect of the cooling rate on the mechanical properties of glass fiber reinforced thermoplastic composites. Compos. Struct..

[33] Zhang J.D., An P., Yu K., Liu G., Huang J., Gu Y., Yao J., Liu H., Chen C., Zhang C. (2023). The effect of cooling rates on crystallization and low-velocity impact behaviour of carbon fibre reinforced poly(aryl ether ketone) composites. Compos. Part B—Eng..

[34] Gao S.L., Kim J.K. (2001). Cooling rate influences in carbon fibre/PEEK composites. Part II: Interlaminar fracture toughness. Compos. Part A—Appl. S..

